# Characterization of pH-responsive high molecular-weight chitosan/poly (vinyl alcohol) hydrogel prepared by gamma irradiation for localizing drug release

**DOI:** 10.1186/s40643-022-00576-6

**Published:** 2022-08-27

**Authors:** Tu Minh Tran Vo, Thananchai Piroonpan, Charasphat Preuksarattanawut, Takaomi Kobayashi, Pranut Potiyaraj

**Affiliations:** 1 Department of Materials Science, Faculty of Science, Chulalongkorn University, Bangkok, 10330 Thailand; 2 Department of Energy and Environment Science, Nagaoka University of Technology, Kamitomioka, 1603-1, Nagaoka, Niigata 940-2188 Japan; 3 Center of Radiation Processing for Polymer Modification and Nanotechnology (CRPN), Faculty of Science, Kasetsart University, 50 Ngam Wong Wan Rd, Lat Yao, Bangkok, 10900 Chatuchak Thailand; 4 Department of Metallurgical Engineering, Faculty of Engineering, Chulalongkorn University, Bangkok, 10330 Thailand; 5 Center of Excellence on Petrochemical and Materials Technology, Chulalongkorn University, Bangkok, 10330 Thailand; 6 Center of Excellence in Responsive Wearable Materials, Chulalongkorn University, Bangkok, 10330 Thailand

**Keywords:** Chitosan, Poly(vinyl alcohol), Gamma irradiation, Targeted drug release

## Abstract

pH-sensitive hydrogels prepared by gamma irradiation find promising biological applications, partially, in the field of localized drug liberation. Herein, optimal conditions for fabricating high-molecular-weight chitosan/polyvinyl alcohol hybrid hydrogels using gamma irradiation at 10, 25, and 30 kGy were investigated by studying the water uptake behavior, the pore size on the surface, and thermal stability. Furthermore, the crosslinking mechanism of irradiated hydrogels was examined via solid-state ^13^C NMR spectrum. The swelling ratio of the gamma-irradiated CS/PVA hydrogel was pH-dependent; particularly, the hybrid hydrogel exhibited high swelling ratios under acidic conditions than those under basic conditions due to the protonation of amino groups on CS-backbone in acidic environments. In addition, amoxicillin was used as a model drug in the in vitro drug release investigations in pH-simulated gastric fluid and deionized water at 37 °C. To identify the drug release mechanism, several kinetic models composing zero-order, first-order, Higuchi, Hixson–Crowell, and Korsmeyer–Peppas models were used. The findings suggested that drug release is mediated by a non-Fickian transport mechanism.

**Graphical Abstract:**

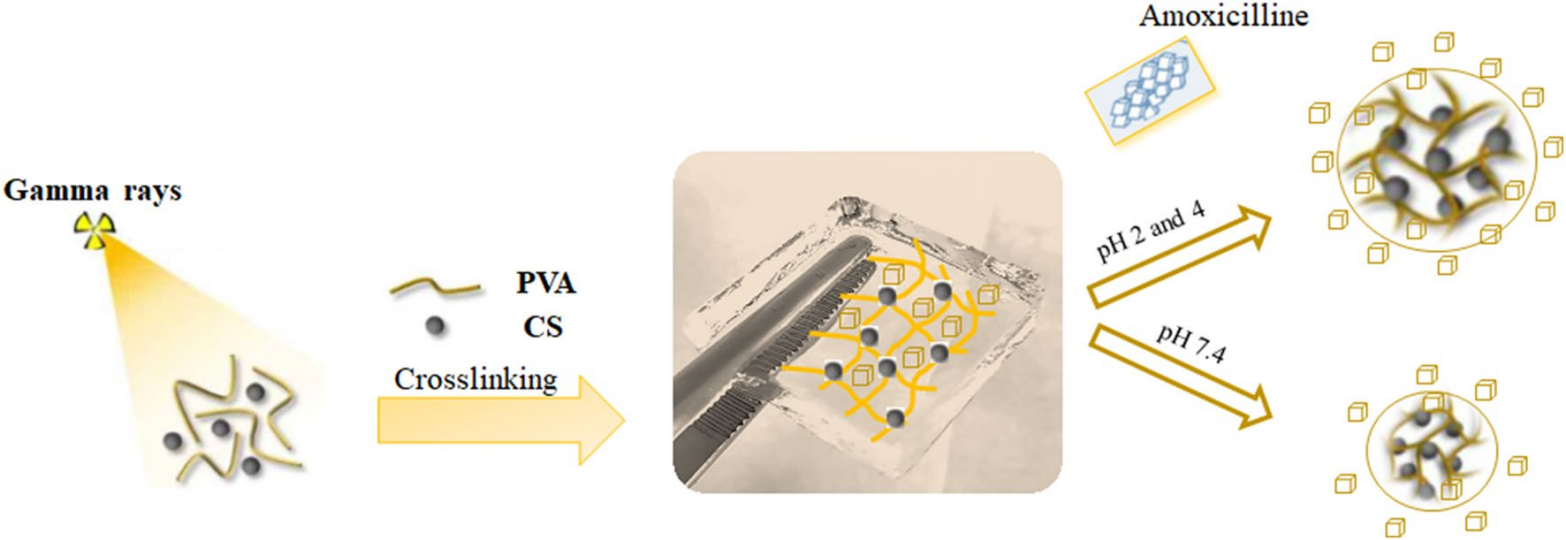

## Introduction

A hydrogel is a three-dimensional (3D) network of polymers; it possesses the unique ability to absorb a large amount of water or biological fluids which arises from the presence of hydrophilic groups on the polymer backbone (Ahmed 2015). Hydrogels are promising materials for biomedical applications owing to their various properties including permeation and swelling behaviors, and surface, optical, and mechanical properties, which can be tuned by changing the chemical composition of the hydrogels or controlling the blending conditions, the crosslinking strategy, or the crosslink density (Ullah et al. 2015). Interestingly, pH-responsive smart hydrogels are extensively used in biomedical applications, because their swelling and deswelling behaviors are the unique requirements for targeted drug delivery. In detail, with a change in the pH or ionic strength of the solution, the volume of a hydrogel varies due to interactions between H bonds and ions. This phenomenon is attributed to bond dissociation and electrostatic repulsion, resulting in swelling and shrinking behaviors (Chaves et al. 2019).

Amoxicillin is an effective agent for treating Helicobacter pylori infections, the main factor causing stomachache. The drawbacks of traditional therapies may be related to the low permeabilities of antibiotics. To improve this obstacle, hydrogels are employed in the formation of controlled and mucoadhesive drug delivery systems because of their hydrophilicity and network structures, which facilitate the encapsulation of drugs and regulation of drug release. Hydrogels with controllable swelling and crosslinking behaviors may be advantageous as possible drug carriers (Zhao et al. 2020). Moreover, one of the most significant benefits of these hydrogels is that they remain in the target region for a longer period than traditional hydrogels. To improve drug loading efficiencies, polymers have been incorporated into hydrogels, and expected results have been acquired, indicating that the introduction of a polymeric network directly influences the drug absorption and release behaviors of hydrogels (Altinisik and Yurdakoc 2014).

Crosslinked-biomedical materials are the ongoing revolution in scientific innovations that bridge diverse areas from physics, chemistry, and medicine, thereby laying the foundation of technological implementation. These upcoming materials are still a fully unexplored sector to be examined by scientists. Crosslinked-biomedical materials have been extensively investigated. Among the numerous crosslinking techniques available, physical and chemical crosslinking are 2 fundamental methods (Wang et al. 2019). Chemically crosslinked hydrogels can be synthesized by several methods, such as “click” chemistry (Fu et al. 2017), enzyme-induced crosslinking (Zhang et al. 2017), photopolymerization (Hu et al. 2016), stiff-base reaction (Ma et al. 2018), and irradiation methods (electron beam irradiation (Dehghan-Niri et al. 2020), UV irradiation (Xu et al. 2018), and gamma irradiation (Islam et al. 2014). Chemical crosslinking has been widely applied for the preparation of hydrogels; nevertheless, significantly high toxicities of crosslinking agents have been noticed. Hence, the development of a green method for the fabrication of hydrogels for biomedical applications is fundamentally necessary. Thus, a simple, effective, environmentally friendly, and productive irradiation method is proposed. Furthermore, crosslinking is performed using gamma irradiation at ambient temperature to solve the issue of overheating in the case of other methods and involves radiation sterilization simultaneously. Crosslinking or degradation may dominate up to the radical position on its backbone. Crosslinking is caused by the recombination of radicals produced in the side chain, whereas degradation is induced by the recombination of radicals formed at the glycosidic bond (Ashfaq et al. 2020).

Chitosan (CS), the second most abundant polysaccharide after cellulose, is a derivative of chitin, the main component of the exoskeletons of insects and shellfish and the cell walls of fungi (Yadav et al. 2019). CS comprises glucosamine and N-acetylglucosamine units joined together by β-1, 4-glycosidic linkages (Ramawat and Mérillon 2015). Depending on the presence of aliphatic (–CH), ether (–C–O–C), and hydroxyl (–OH) groups of primary alcohol and numerous basic amino (amide, –NH) groups, hydrogels can exhibit cationic polyelectrolyte properties and act as ion exchangers. Owing to the existence of NH_2_ and OH groups found on their molecules, adherent polysaccharides are highly mucoadhesive, contributing to their long-term stay in the gastrointestinal system. CS is an excellent excipient due to its non-toxicity, biodegradability, and pH dependence; thus, it is an ideal candidate for controlled release formulations (Singh and Dutta 2011). However, pure CS undergoes degradation primarily because of chain scission rather than crosslinking under ionizing radiation, reducing the viscosity of the solution (Hayrabolulu et al. 2014). Furthermore, in vitro and in vivo biomedical applications of CS are limited by its inferior thermal stability, instability of its main structure, and lack of mechanical strength. To overcome the limitations of pure CS, materialization methods to reinforce its structure while retaining its biocompatibility need to be developed, considering the significant importance of high-performance materials produced from natural sources in biomedicine. To considerably increase thermomechanical stability, antibacterial characteristics, and lowered water evaporation of hydrogel, Yang and partners crosslinked the PVA/CS hydrogel membrane (Yang et al. 2008). In contrast, synthetic polymers are easily characterizable and reproducible. The majority of synthetic polymers are formed by covalent crosslinking, which contributes to the mechanical and thermal properties of these polymers (Yu et al. 2006). PVA is a water-soluble synthetic polymer and possesses many advantages, for example, biocompatibility, crosslinking ability, and non-toxicity; thus, it does not cause any toxicity in the body (Stammen et al. 2001). Therefore, PVA has been utilized as a reinforcer for collagen hydrogel in biomedical applications, and the highest cell proliferation has been achieved at a collagen/PVA ratio of 1:1 (Zhou et al. 2020). Physical properties, including water-absorption properties and gelation behavior, of a gamma-irradiated CS/PVA hydrogel, were enhanced by increasing the radiation doses, and the water vapor transmission rate increased from 50 to 70%, implying satisfactory properties of this blended hydrogel for wound dressing (El Salmawi 2007).

In this study, we optimized the parameters for the fabrication of CS/PVA hybrid hydrogels by gamma irradiation. In the hybrid hydrogels, high molecular-weight (*M*_w_) CS was examined, because the rapid degradation of CS-based hydrogels with low molar masses. Furthermore, gamma crosslinking is affected by polymer morphology, with the amorphous phase forming a larger crosslinked network than the crystalline phase and high Mw of CS leads to the rising amorphous area (Naikwadi et al. 2022). Influences of the composition of CS, PVA, and radiation doses on the physical properties. The formation of free radicals on CS/PVA and the mechanism of crosslinking under irradiation were examined via ^13^C-nuclear magnetic resonance (^13^C-NMR) spectroscopy. Scanning electron microscopy (SEM) images explored the pore size of hydrogel morphology. In addition, the stabilities of the blended CS/PVA hydrogels were higher than that of pure CS, and the swelling and shrinking behaviors of these pH-responsive hydrogels were dependent on the charges of the pH-responsive polymers. Therefore, stimuli-responsive CS/PVA hydrogels can be applied in drug delivery systems. Herein, pH-responsive swelling kinetics of the hydrogels were analyzed to investigate the prospective applications of stimuli-responsive hydrogels in the localized release of amoxicillin. To better understand the drug release process, the release profiles were examined using several models.

## Materials and methods

### Materials

High molecular-weight (*M*_w_) CS (*M*_w_ of 700 kDa and degree of deacetylation of 80%) was provided by Seafresh Chitosan (Lab) Co., Ltd., Thailand. PVA (*M*_w_ of 145,000 g/mol) was purchased from CT Chemicals Ltd., Thailand, and glacial acetic acid was obtained from Merck Co., Ltd., Germany. Amoxicillin trihydrate (Ibiamox) was procured from Siam Bheasach Co., Ltd., Thailand. All other reagents used herein were of analytical grade.

### Synthesis of hybrid hydrogels

In the first step, high-molecular-weight CS powder was dispersed in 1% acetic acid solution at room temperature, and PVA was dissolved in DI water at 90 °C to obtain a visually clear solution with 1.5% of CS and 5% of PVA. Second, CS/PVA at different ratios (100/0, 25/75. 50/50, 75/25, and 0/100) were intermingled to achieve homogeneous polymer blend solutions. Note that CS solution at higher concentration could not be synthesized due to its high viscosity. At last, final blended solutions (6 g) were added to 2.5 × 2.5 cm containers with lids and tightly sealed. These samples were irradiated with gamma rays at doses of 10, 25, and 30 kGy with a dose rate of 1.04 kGy/h using a ^60^Co gamma MARK I irradiator.

### Determination of gel contents

After irradiation, the hydrogels were dried at 60 °C for 48 h and accurately weighed (*W*_*o*_) by a four-decimal electronic analytical balance. To eliminate un-crosslinked fractions, the dried hydrogels were immersed in DI water for 24 h and then dried in a vacuum oven at 60 °C to a constant weight (*W*_*1*_). The experiments were performed with three replicates. The gel fraction was calculated according to the following equation:

1 $${\text{Gel fraction }}\left( \% \right) \, = \, \left( {W_{1} /W_{o} } \right) \times 100$$

### Swelling behavior

The swelling ratio (SR) provides information about the water-absorption capacity of a hydrogel. SR was measured in DI water and solutions with different pH values (1, 4, 7, 10, and 13). Weighed dry samples were immersed in water at room temperature until they swelled to equilibrium. After the surface water was removed with filter paper, the fully swollen samples were weighed. SR was calculated using the following equation:

2 $${\text{SR }} = \, \left( {W_{{\text{s}}} {-} \, W_{{\text{d}}} } \right)/W_{{\text{d}}}$$

where *W*_s_ is the weight of the swollen sample at equilibrium and *W*_d_ is the weight of the dry sample. Experiments were conducted in triplicate.

### Swelling kinetic studies under different pH values

The most basic Fick’s law is used for explaining the swelling kinetics and diffusion of polymeric structures. The swelling kinetics of polymers can be presented by the following equation:

3 $$F = \, W_{{\text{t}}} /W_{{\text{e}}} = Kt^{n}$$

where *W*_t_ and *W*_e_ represent the amount of water absorbed by the hydrogel at a time t (seconds) and equilibrium, *K* is a constant characteristic of the structure of the networks and *n* is an exponent that determines the mode of water diffusion. When *ln F* is plotted against *ln t*, it gives a straight line from which the intercept determines the constant *K* and the slope gives the number *n*. In this regard, a value of *n* = 0.5 indicates a Fickian diffusion mechanism in which the sorption is diffusion controlled, whereas a value of 0.5 < *n* < 1 indicates an anomalous non-Fickian type diffusion and contributes to the water-sorption process (Demeter et al. 2017; Ganji et al. 2010).

### Confirmation of the chemical structure

Chemical structures of the hydrogels were investigated by ATR–FTIR spectroscopy using a Nicolet™ iS50 FTIR Spectrometer in the 4000–500 cm^−1^ range with a resolution of 4 cm^−1^, and an average of 32 scans were acquired. The analysis was performed in triplicate. ^13^C Magic-angle spinning nuclear magnetic resonance (^13^C MAS NMR) spectra of neat CS, pure PVA, and CS/PVA gel beads were acquired using an AVANCE III 400 WB spectrometer (Bruker, Billerica, MA, USA) operated at a frequency of 400 MHz to explore the chemical structures of the crosslinked hydrogels.

### Scanning electron microscopy (SEM)

To observe the morphologies and the natures of the hydrogel networks, the structure of the freeze-dried samples were investigated by SEM (Hitachi SU5000 with a magnification of 1000 ×) using the Au-coated hydrogel samples (5 mm × 5 mm) fabricated.

### Thermogravimetric analysis (TGA)

Thermal analysis of hydrogels was conducted using a thermogravimetric analyzer (TGA/DSC3þ STARe System, Mettler Toledo, Columbus, OH, USA). The samples (approximately 5 mg) were heated from 50 to 600 °C at a heating rate of 10 °C/min under an N_2_ atmosphere. Maximum thermal degradation temperature was evaluated by thermal mass loss (TG) and derivative thermogravimetric (DTG) data.

### In vitro* drug release test*

Model amoxicillin drug was loaded in CS/PVA irradiated hydrogel by the post-loading method. First, the hydrogel was immersed in a 1 mg/ml model drug solution at room temperature for 24 h to fully swell. The amoxicillin-loaded hydrogels were placed in the oven for 24 h at 37 °C to completely dry. Second, the dried hydrogels were then weighted and the drug uptake was examined using Peppas’s equation (4). The amount of drug uptake was calculated from the standard curve:

4 $${\text{Drug uptake }} = {\text{The amount of amoxicillin}} - {\text{Loaded hydrogel}}/{\text{The initial weight of hydrogel }}\left( {\mu {\text{g}}/{\text{mg}}} \right)$$

Releases of drugs from these polymer hydrogels in buffer solutions with pH 2.1 and 7.4; in DI were investigated in vitro. Drug-loaded hydrogels were submerged in 10 ml buffer solutions at 37 °C, and drug release was measured. At present time intervals, 3 ml aliquots of physiological media were removed, and the volumes of the media with the released drug were maintained by adding appropriate buffer solutions (3 ml). The contents of amoxicillin in the media were evaluated using a UV–Vis spectrophotometer (UV 9100 Series, LabTech, Hopkinton, MA, USA) at 272 nm based on the calibration curve of Amoxicillin (Fig. 7a). The findings are reported in terms of drug release as a function of time. At the end of this experiment, the hydrogels were removed from the drug-release system and dried in the vacuum oven at 37 °C. Trials were conducted 3 times, and the average results were calculated:

$${\text{Drug release }}\left( \% \right) = \left( {{\text{C}}_{{\text{t}}} /{\text{C}}_{0} } \right){\text{ x 1}}00$$

where C_t_ is the quantity of amoxicillin released from hydrogels at determined time intervals C_0_ is the amount of amoxicillin absorbed into hydrogels.

The following mathematical models were used to investigate drug release kinetics from hydrogel networks (Paarakh et al. 2019):

$${\text{Zero order}}:{\text{ C}}_{t} /C_{0} = k_{0} t$$

$${\text{First order}}:{\text{ log C}}_{t} /C_{0} = - k_{{1}} t/{2}.{3}0{3}$$

$${\text{Higuchi}}:{\text{ C}}_{t} /C_{0} = k_{{\text{h}}} t^{{0.{5}}}$$

$$\begin{gathered} {\text{Korsmeyer}} - {\text{Peppas}}:{\text{ log }}\left( {C_{t} /C_{0} } \right) \, = {\text{log}}\left( k \right) \, + \, n{\text{ log}}\left( t \right) \hfill \\ \hfill \\ \end{gathered}$$

$$\begin{gathered} {\text{Hixson}} - {\text{Crowell}}: \, C_{{\text{o}}}^{{{1}/{3}}} {-} \, C_{t}^{{{1}/{3}}} = k_{{{\text{hc}}}} t \hfill \\ \hfill \\ \end{gathered}$$

where *C*_*t*_ denotes the quantity of drug released at time *t*; *C*_*0*_ defines the initial concentration of drug in the hydrogel; *k*_0_, *k*_1_, *k*_h_, *k*_hc_ and k are the release rate constant, and n is the release exponent which indicates the release mechanism.

### Statistical analysis

A one-way analysis of variance was used to determine the statistical significance of the groups (ANOVA). All data is presented in triplicate, and the mean standard deviation (SD) is computed with the level of significance set at *p* < 0.05 using Origin 2018 software.

## Results and discussion

### Gel fraction

**Fig. 1:**
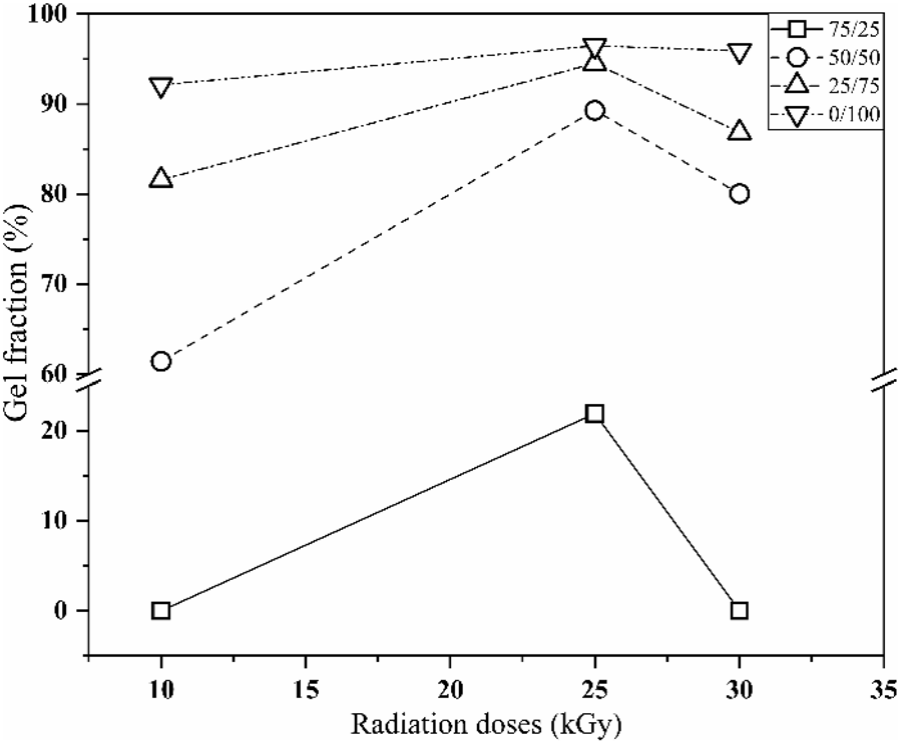
Effect of irradiation dose on the gel fraction (%) of CS/PVA at different compositions

Gel fraction analysis was performed to determine the degree of crosslinking of hydrogels produced by irradiation. The effects of different radiation doses and CS/PVA ratios on the gel fractions of hydrogels are shown in Fig. 1. With an increase in the radiation dose, the gel fraction increased until it reached a maximum at 25 kGy, after which it started to decrease owing to the predominance of chain scission over crosslinking (Tan et al. 2021). At 10 kGy and 30 kGy, 75/25 CS/PVA could not form a gel; meanwhile, at 25 kGy, approximately 20% gel fraction was attained. The gel fraction of the 50/50 CS/PVA hydrogel exhibited significant change greater than 4 times as compared to those of the 75/25 CS/PVA hydrogel at 25 kGy. The fraction of the insoluble part increased with an increase in the PVA content in the composite hydrogel due to the increase in the number of PVA radicals to form 3D networks. Thus, crosslinking predominated degradation. Nevertheless, the gel fraction substantially decreased with an increase in the CS content, because a higher amount of CS as compared to that of PVA hindered radical recombination. Consequently, no crosslinking occurred. In addition, water in the polymer solution played a crucial role in realizing maximum crosslinking, resulting in a higher number of free radicals, thereby improving the generation of macroradicals. Furthermore, H atoms and –OH radicals can produce additional macroradicals by eliminating H from PVA molecules (Jeon et al. 2018). During irradiation, radical crosslinking and chain scission are two main reactions that simultaneously occur.

### Swelling behavior

The ability of a hydrogel to retain water/biological fluid is a critical factor in determining the suitability of the hydrogel for biomaterial applications. The hydrogel experiences a change in swelling rate in a selected pH environment for an excellent tailored drug release.

**Fig. 2:**
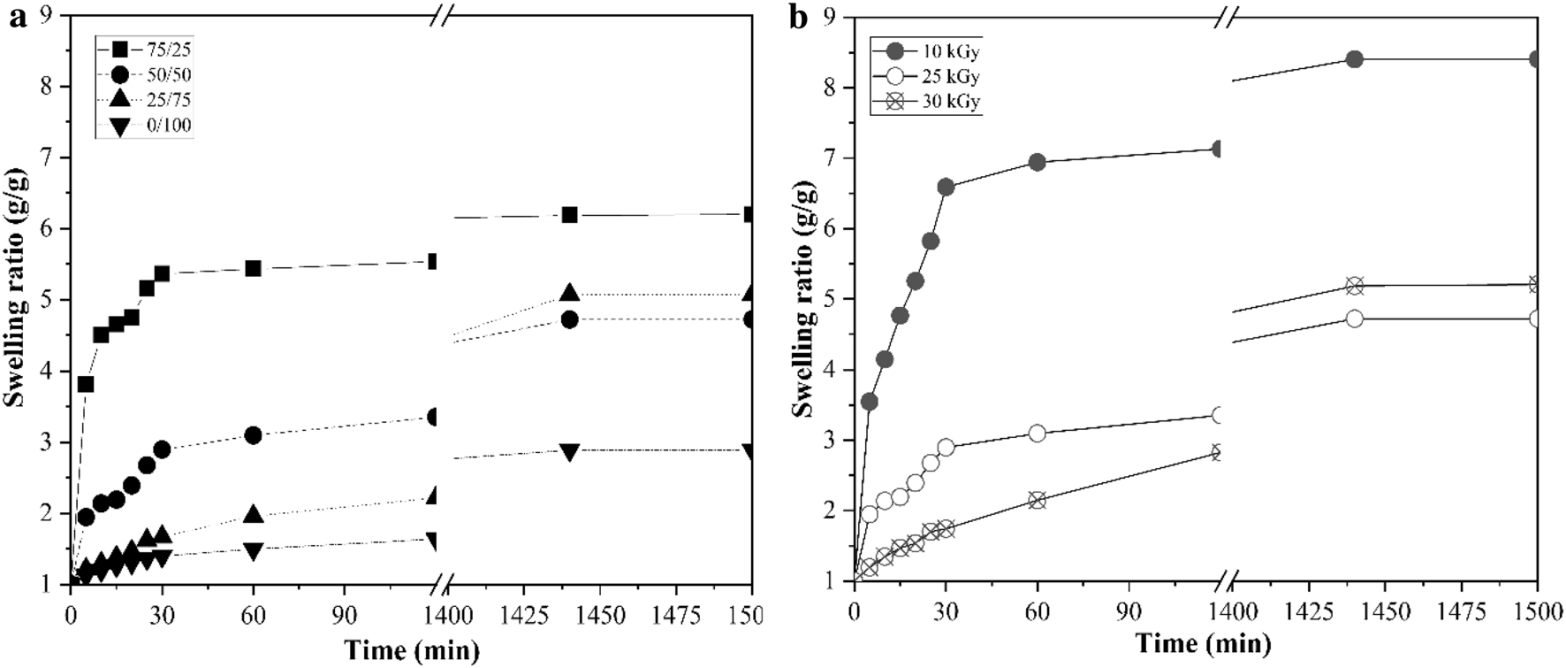
Swelling ratio (g/g) vs. time (min) graph of the prepared hydrogels in deionized water at different polymer ratios **a** and radiation doses of 50/50 CS/PVA hydrogel **b**

In DI water, crosslink density is primarily responsible for the swelling abilities of hydrogels. The SRs of hydrogels at different radiation doses and CS/PVA content as a function of time are depicted in Fig. 2. Hydrogels prepared at 10 kGy swelled at different rates when compared with those of the hydrogels fabricated at higher radiation doses (25 and 30 kGy). At 10, 25, and 30 kGy, the equilibrium swelling degrees of the 50/50 CS/PVA hydrogel were 8.4-, 4.7-, and 5.2-fold compared to the dried state after 24 h, respectively. In contrast to the case at 10 kGy, the higher crosslinking of polymer chains at 25 kGy formed a stronger network with higher resistance to expansion, thereby reducing the swelling degree. However, owing to predictable polymer chain scission and a resultant decrease in the crosslinking density, the swelling degree of the hydrogel generated at 30 kGy slightly increased when compared with that of the hydrogel produced at 25 kGy. The amount of absorbed water in the gel network considerably increased before reaching a plateau. Moreover, the SR decreased with an increase in the PVA content, because with an increase in the PVA concentration of hydrogels, the crosslink density increased, thereby decreasing the SR.

### Swelling indices in different pH solutions

**Fig. 3:**
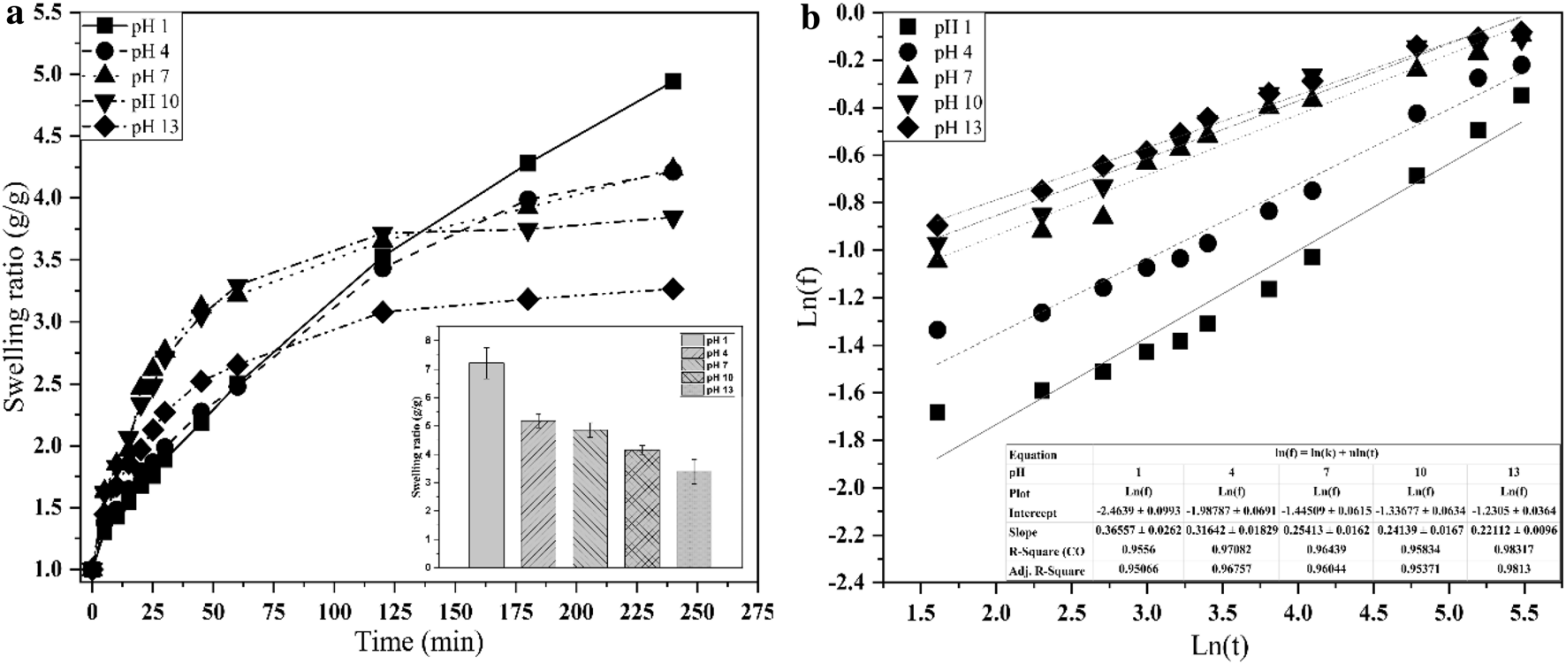
Swelling ratios of the 50/50 CS/PVA hydrogel discs at **a** pH 1–13, **b** ln(*f*) vs. ln(*t*) plot

The swelling behavior of hydrogels is a complicated phenomenon that involves three consecutive steps: diffusion of the solvent into the network, chain relaxation inside the hydrated gels, and network expansion. Functional groups lead to electrostatic repulsion inside the network, thus expanding the gel and ultimately resulting in equilibrium. The presence of more free amino groups in the network leads to stronger electrostatic repulsion between polymer chains and a faster swelling rate (Li 2009). According to the findings of this study, the pH sensitivity of the hydrogel originates from the different swelling degrees of the hydrogel in buffer solutions with different pH values. At pH 7, 10, and 13, the 50/50 CS/PVA hydrogel prepared at 25 kGy had a lower SR than those at pH 1 and 4. The swelling degree of the hydrogel at pH 1 increased by twofold to approximately 7 (g/g) compared to that at pH 13. Furthermore, the ionic strength of the solution may influence the swelling degree. High M_w_ CS provides a better possibility for crosslinking either chemically or physically which forms a 3D network structure by increasing the entanglement to reduce repulsive force which tends to increase intramolecular interaction at pH 5.5–6 of CS/PVA blended hydrogel. The amino groups of high M_w_ CS can be protonated (NH^3+^) in acidic fluids at low pH, and the electrostatic repulsions induced by these ionic groups can increase the hydrophilicity of the hydrogel, thereby expanding the hydrogel networks. In contrast, swelling decreases under neutral (pH 7) and alkaline (pH 10 and 13) conditions due to the deprotonation of these amino groups. Owing to the increasing demand for controlled drug delivery with high accuracy, the pH-sensitive swelling behaviors of hydrogels may be advantageous for regulating drug release (Almáši et al. 2020; Ding et al. 2021; Fan et al. 2019; Yang et al. 2008). During swelling, water molecules invade the hydrogel surface and diffuse inside. Fick’s law is employed to explain the process by which the water molecules diffuse through swollen materials, such as hydrogels. Fick’s laws are depicted in Fig. 3, and the values of *k* and *n* are presented in Table 1. For all CS/PVA samples, the estimated *n* values ranged between 0.2211 and 0.3556, which were all less than 0.5 for the hydrogels investigated herein, which was consistent with the previously reported results (Wang et al. 2008). With an increase in pH, the mobilities of polymer chains reduced; simultaneously, the affinity between the water molecules and the polymer substantially decreased, resulting in a sluggish rate of diffusion of water molecules into the gel; therefore, the *n* values decreased (Wang et al. 2008). The *n* values implied that all the CS/PVA samples exhibit Fickian water transport. CS/PVA samples with minimal swelling demonstrate Fickian (less relaxation-controlled) behaviors, because hydrogel ionization is dominant and the H bond regulates solvent transport. The ionization of functional groups affects the mechanism of water diffusion, influencing both the relative magnitude of diffusion and the swelling degree (Ghobashy et al. 2021).

**Table 1:** Diffusion parameters of the 50/50 CS/PVA hydrogel at different pH values

Sample	Intercept	*k*	*n*	*r*^2^
pH 1	− 2.4639	0.0851	0.3556	0.9556
pH 4	− 1.9879	0.137	0.3164	0.9708
pH 7	− 1.4451	0.2357	0.2541	0.9644
pH 10	− 1.3368	0.2629	0.2414	0.9583
pH 13	− 1.2305	0.2921	0.2211	0.9832

### Chemical structures of crosslinked CS hydrogels

**Fig. 4:**
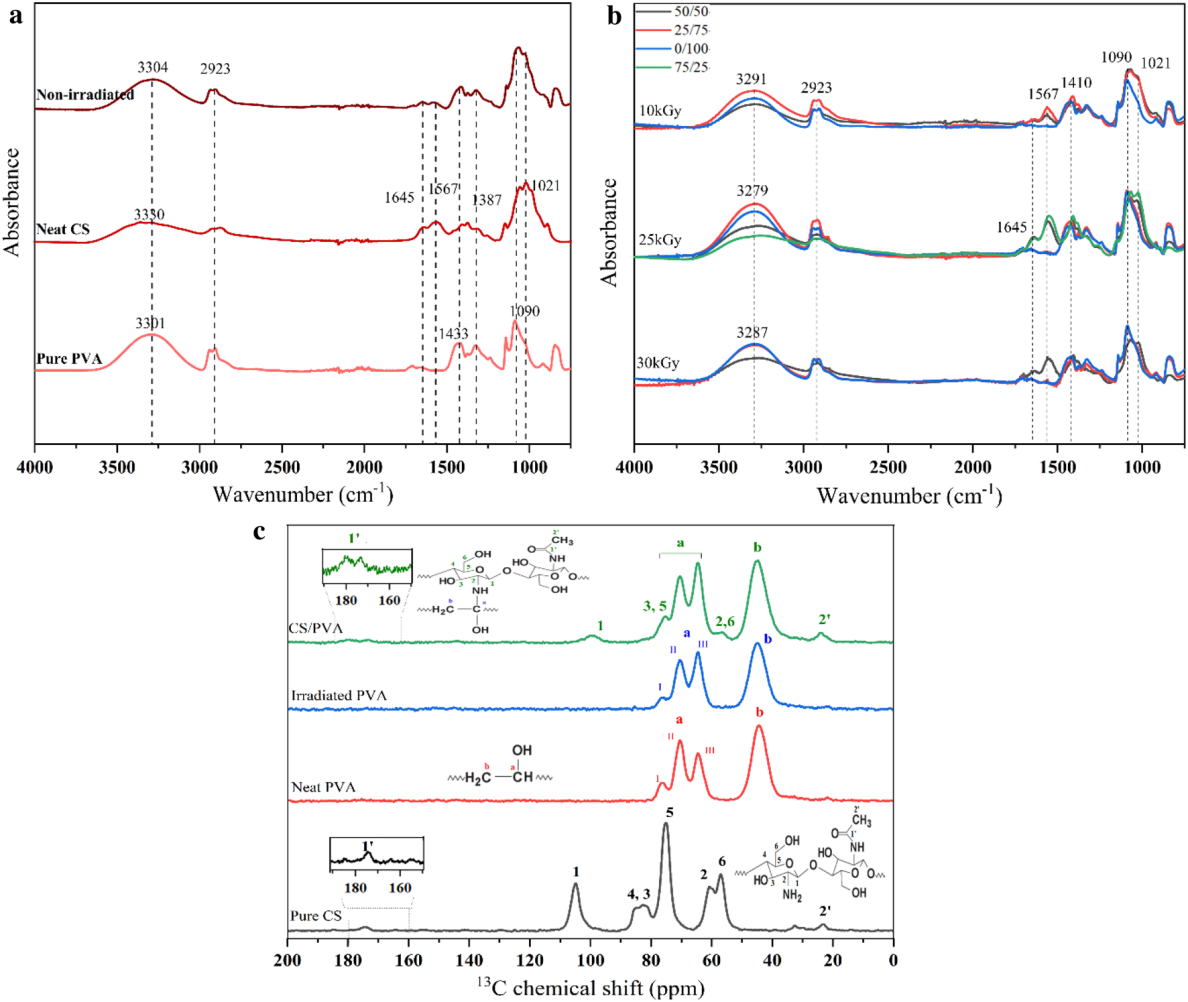
**a** Fourier transform infrared spectra of pure PVA, neat CS, non-irradiated CS/PVA, and **b** FTIR of crosslinked hydrogels irradiated at 10, 25, and 30 kGy; **c** solid-state 13C nuclear magnetic resonance spectrum of pure CS, neat PVA before and after irradiation, and the 50/50 CS/PVA hydrogel

Distinctive functional groups and newly formed bonds between neat CS, pure PVA, and CS/PVA hybrid hydrogels were assessed via FTIR absorption spectroscopy and solid-state ^13^C NMR spectroscopy. FTIR spectra of unirradiated and gamma-irradiated CS/PVA hydrogels are shown in Fig. 4.

FTIR spectrum of pure PVA (Fig. 4a) shows a broad absorption band at 3301 cm^−1^, corresponding to the stretching and bending vibrations of the –OH group. The peaks at 2923, 1433, and 1090 cm^−1^ are ascribed to the C–H stretching of alkyl groups, C–H bending, and C–O group stretching, respectively (Abureesh et al. 2016). The peak of neat CS at 3330 cm^−1^ is attributed to the vibrational stretching of the N–H and O–H intermolecular and intramolecular H bonds. Stretching vibration absorption peaks of C–H on the CS chain were observed at approximately 2879 cm^−1^. Moreover, the absorption peaks of CS were noticed at 1645 (amide I), 1567 (amide II), and 1387 cm^−1^ (amide III), which primarily originated from the stretching vibration of C–O, the vibration of the N–H bond, and the stretching vibration of the C–N bond, respectively (Kong & Yu 2007). Peaks related to the antisymmetric stretching of the C–O–C bridge and C–O vibration of the ring, which are characteristic peaks of the saccharide backbone, were observed at 1141 and 1021 cm^−1^, respectively (Bisen et al. 2017). FTIR spectrum of the hybrid hydrogel exhibits all the characteristic peaks of both CS and PVA hydrogels. The peaks of the non-irradiated samples shifted from 3304 to 3291, 3279, and 3287 cm^−1^ compared to those of the irradiated samples because of the formation of an intermolecular H bond between CS and PVA. This H bond functions as a connector between the two polymers. In addition, the obtained data indicated that no substantial degradation of CS occurred in the case of the 50/50 hydrogel samples irradiated at different radiation doses (Fig. 4b) as the intensity of the peak at 1567 cm^−1^ had slightly increased (Mozafari et al. 2012; Bisen et al. 2017; Casimiro et al. 2021; Casimiro et al. 2021).

Chemical structures of hydrogels were verified by solid-state ^13^C NMR spectroscopy. Table 2 presents the integrals of the NMR resonances of the particular functional groups discovered in pure CS, before and after irradiation of neat PVA, irradiated CS/PVA hydrogel. The corresponding assignments are as follows: 0–49 ppm: alkyl C; 49–62 ppm: N–CH; 62–94 ppm: O-alkyl C; 94–110 ppm: O–C–O anomeric C; and 160–188 ppm: COO and N–C–O (Duarte et al. 2020). ^13^C MAS NMR spectra clearly show the peak of methylene C (–CH_2_–) at 44.80 ppm and methine C (– CH–) resonances at 64.53, 70.48, and 75.32 ppm. The methylene carbon is responsible for a well-separated peak at 44 ppm in the both non-irradiated and irradiated PVA spectrum. Carbon connected to OH groups may be allocated to the peaks at 64, 70, and 76 ppm (Jayasekara et al. 2004). Peaks I and II have been ascribed to the isotactic structure with two intramolecular H bonds and the heterotactic structure with one intramolecular H bond, respectively, whereas Peak III has been assigned to the syndiotactic structure with no intervening intramolecular H bonds. Comparing neat PVA before and after irradiation spectra to those of the equivalent dry gels demonstrates that the gelation process fragments the network of the intramolecular hydrogen bonds based on previously reported NMR studies on PVA (Padavan et al. 2011; Lai et al. 2002). In blended hydrogel (Fig. 4c), the combination of PVA and CS peaks was obtained. In addition, the C3 and C5 peaks at 75.32 ppm were two overlapping peaks, which arose from the intramolecular H bond (Wang et al. 2019). C peaks were observed in the spectrum of pure CS (C = O: 174 ppm; C1: 104 ppm; C4/C3: 85–82 ppm; C5: 75 ppm; C2: 60; C6: 58 ppm; and CH_3_: 23 ppm). Herein, the C1 peak showed minor low-field shifts after the irradiation of the hybrid hydrogel as compared to the case of pure CS and intramolecular/intermolecular H bonds formed around C2, C3, C5, and C6 during crosslinking, resulting in slight low-field shifts of the corresponding peaks, which were in agreement with the findings reported in the literature (Yang et al. 2021; Heux et al. 2000).

**Table 2:** Chemical shifts of C in the pure and blended hydrogels

Chemical shift (ppm)	Assignment	References
23.96	− CH_2_– carbon of residual acetate	(Jayasekara et al. 2004)
44.80	− CH_2_–	(Jayasekara et al. 2004)
56.54	C2	(Jayasekara et al. 2004)
64.53	C6, Peak I	(Jayasekara et al. 2004; Lai et al. 2002)
70.48	Peak II	(Lai et al. 2002)
75.32	Peak I	(Lai et al. 2002)
99.98	C1	
173.20	C=O (ester)	(Katoh and Ando 2016)
180.61	C=O (amide)	(Katoh and Ando 2016)

**Scheme 1:**
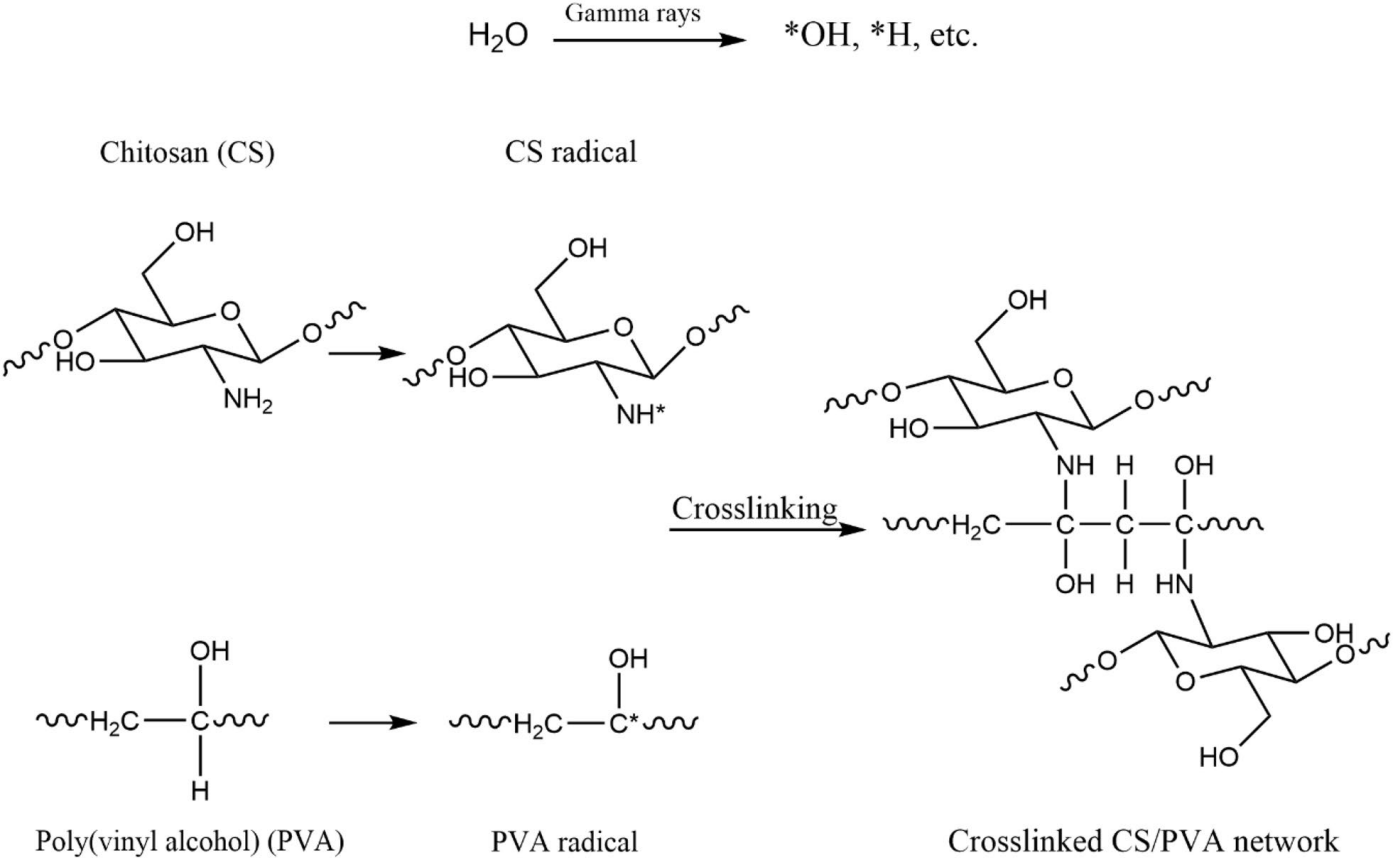
Proposed crosslinking process of CS/PVA hydrogel under gamma irradiation

To elaborate the crosslinking mechanism of CS/PVA hydrogel by gamma irradiation, the proposed crosslinking process of CS/PVA hydrogel under gamma irradiation is shown in Scheme 1. Water in aqueous solutions absorbs the majority of the gamma-radiation energy. Radiolytic products of water are mainly formed by indirect action on water molecules yielding radicals ·OH, e^−^_aq_, and ·H. (Makuuchi and Cheng 2012). The main reactive species is •OH, which readily removes H from polymer chains and causes the production of CS and PVA radicals and water. In the final phase, covalent bonds between the polymer chains are generated by recombining two macro radicals which are PVA–CS, PVA–PVA, and CS–CS radicals.

### Thermal analysis

**Fig. 5:**
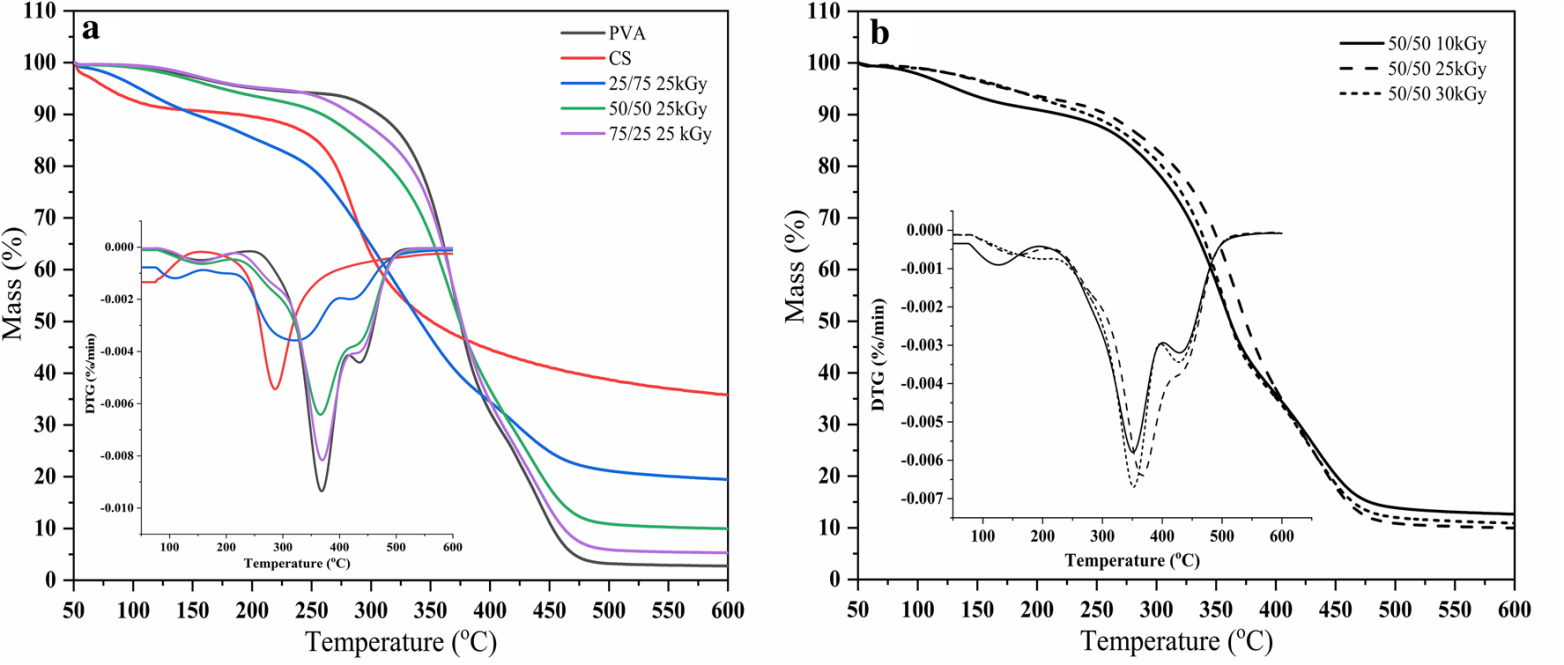
**a** Typical TG and DTG curves of pure PVA, pure CS, and CS/PVA blended hydrogels at 25 kGy and **b** 50/50 CS/PVA at 10, 25, and 30 kGy

TGA was conducted to better understand the thermal characteristics of the hydrogels. Figure 5a shows the weight loss (TG) and derivate (DTG) curves of pure PVA, neat CS, and blended CS/PVA hydrogels with different contents of CS and PVA and at various radiation doses. According to the TGA data, CS deteriorated in two stages. The first signs of degradation appeared at 60 °C, resulting in a 10% weight loss due to the loss of water molecules. Thermal and oxidative degradations of CS were responsible for the subsequent 49% weight loss between 280 and 350 °C. It was caused by the breakdown of the primary components of CS, most notably the heat degradation of the pyranose ring and the fracture of the b-glycosidic bonds connecting the glucosamine and N-acetylglucosamine moieties (Pawlak and Mucha 2003; Martel-Estrada et al. 2014). Moreover, the main decomposition (DTG curve) occurred between 300 and 414 °C and was attributed to the dehydroxylation of PVA, suggesting the initiation of polymeric chain decomposition, and the subsequent decomposition occurred between 414 and 475 °C. This phase involved the continuation of the polyene structure via the generation of C and hydrocarbons (Release 2017). The decomposition temperatures (*T*_d_) of CS, PVA, and blended hydrogels were 223, 267, and 240–266 °C, respectively (Fig. 5a). Thus, PVA had the highest *T*_d_ and was most thermally stable owing to intramolecular and intermolecular H-bonding between its chains. Furthermore, hybrid hydrogels with 50–75% PVA were more thermally stable than those with 25% PVA. This improvement in thermal stability originated from the high degree of crosslinking induced by gamma radiation, which resulted in the formation of a network structure. When the radiation dose was increased from 10 to 30 kGy, the *T*_d_ of the 50/50 CS/PVA hydrogel slightly increased, revealing higher crosslink densities at higher radiation doses (Fig. 5b).

### Morphologies of the crosslinked CS hydrogels

**Fig. 6:**
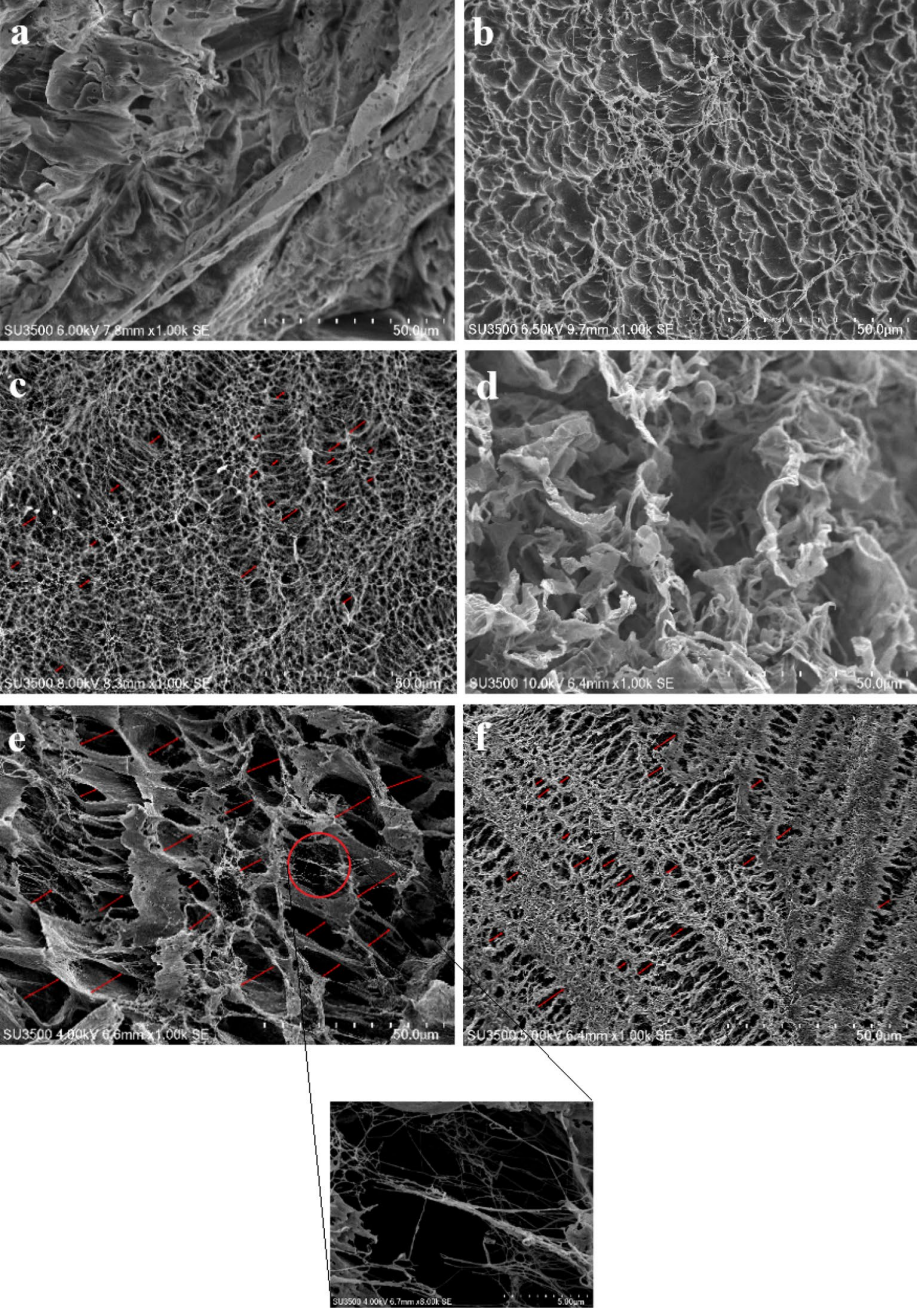
Scanning electron microscopy images of the crosslinked hydrogels:** a** pure PVA, **b**–**d** 25/75, 50/50, and 75/25 CS/PVA-based hydrogels crosslinked at 25 kGy, respectively; **e**, **f** 50/50 CS/PVA-based hydrogel crosslinked at 10 and 30 kGy

For migration, drugs absorb and release in a 3D network; thus, the morphology and interconnectivity between pores are crucial factors in this regard. Topologies of the hydrogel networks with various CS/PVA ratios were examined using SEM. The average diameter of 50/50 CS/PVA hydrogels at 10, 25, and 30 kGy was determined from SEM images employing a magnification of 1000x. Twenty locations were randomly chosen and measured for each sample using ImageJ software. The hydrogel framework obtained at 10 kGy had fewer holes and larger pore sizes of approximately 8.83 µm, indicating limited crosslinking points between polymers. The greater crosslinked network structure was formed when the radiation dose was increased to 25 and 30 kGy. The average pore sizes of the samples decreased to 3.59 µm when the radiation dosage was increased from 10 to 25 kGy and slightly expanded to 4.63 µm at 30 kGy (Fig. 6). With a further increase in the radiation dose, a minor alteration in the porous structure was noticed. With an increase in the PVA concentration, numerous connected chains were generated, reducing the average pore size. Moreover, the morphology of neat PVA, which exhibited an extremely inferior porous pattern, was different from those of the hybrid hydrogels. The porous interpenetrating mesh structure provided excellent permeability, which improved drug transport through the hydrogels. Due to their highly interconnected porous structures forward the moderate pore size, the blended hydrogels may be used for the loading and release of medicines owing to their better swelling characteristics as compared to those of other hydrogels (Vo et al. 2020).

### In vitro* amoxicillin release studies*

**Fig. 7:**
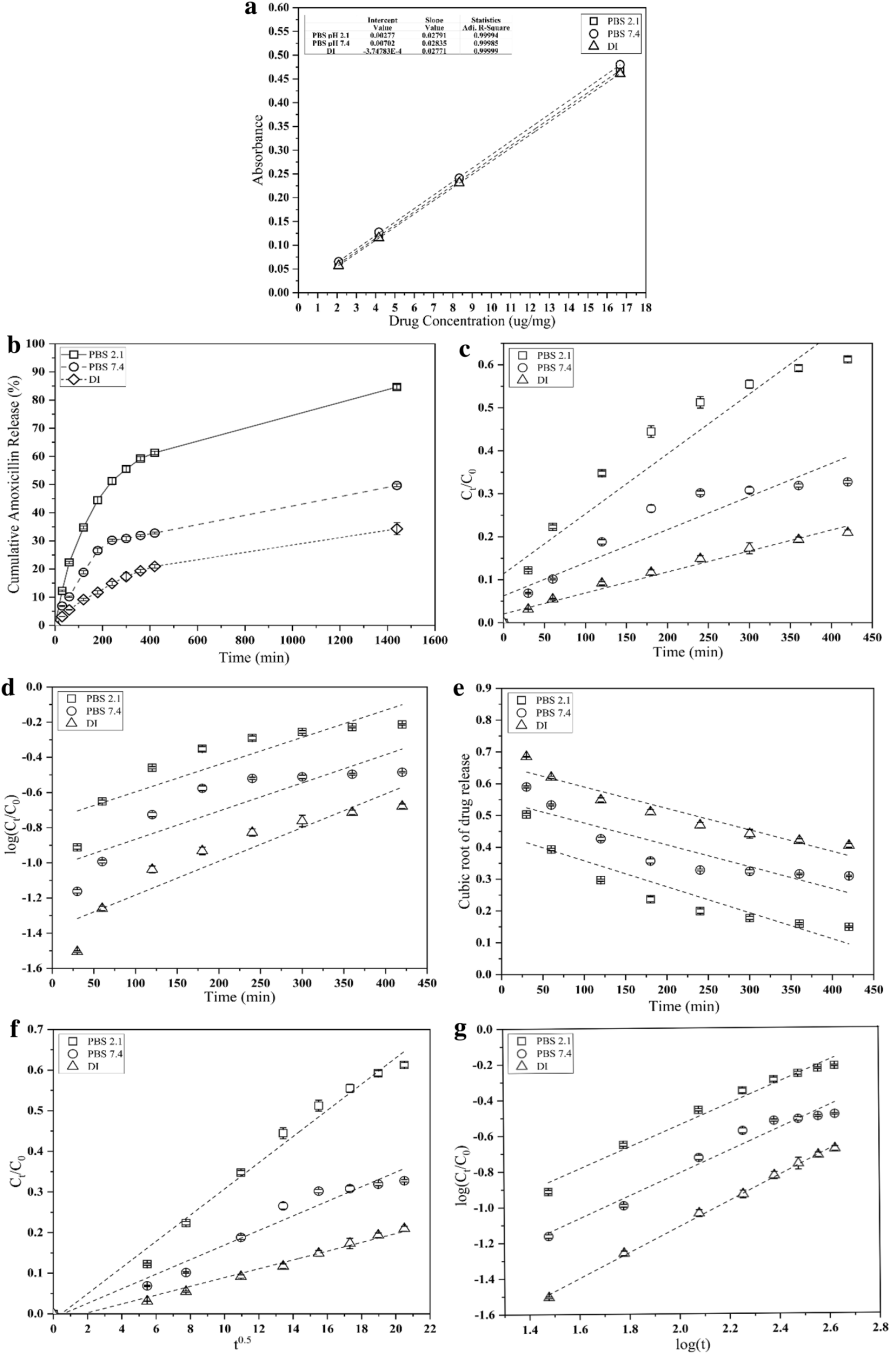
**a** Calibration curve for Amoxicillin using UV-vis spectroscopy, **b** cumulative release of the 50/50 CS/PVA; the drug release profiles of CS/PVA 50/50 were calculated using the **c** zero-order, **d** first-order, **e** Hixson-Crowell model, **f** Higuchi model, and **g** Korsmeyer-Peppas model

The goal of localized release systems is to concentrate medicine concentration in the targeted organ to reduce side effects caused by unfocused release points (Ilgin et al. 2019), Fig. 7a. In this condition, the amount of amoxicillin uptake of CS/PVA gels was 11.133 ± 0.231 µg/mg polymer. At 37 °C, UV spectrophotometry was employed to monitor amoxicillin in vitro release from hydrogel networks over 1440 min following pH changes in PBS medium and DI water. Figure 7b depicts the drug release findings. It is noted that the fast release of amoxicillin in all of the samples at different pH values took place in the first 300 min. The percentage release of amoxicillin from hydrogel at 1440 min was estimated at 85%, 50% at pH 2.1 and 7.4 in PBS media; at 34% at pH 5.5 in DI water. Under acidic conditions, the amount of drug released from the hydrogel increased owing to electrostatic repulsion induced by the protonation of amino groups as this repulsion offered a larger surface area for drug release. Furthermore, the porous structure played a crucial role in drug release as it enhanced the drug permeation ability (Aycan and Alemdar 2018; Constantin et al. 2017; Mulchandani et al. 2017). According to the results, the release of amoxicillin in the physiological environment of PBS is greater compared to in DI water. The higher percentage of released drugs from CS/PVA hydrogel might be due to amoxicillin solubility which is impacted by pH and ionic strength (Palma et al. 2016). Higher ionic strengths were expected to weaken the molecular structure of the polymer by increasing repulsive electrostatic interactions between charged polymer molecules (Vigata et al. 2020).

The use of mathematical modeling to achieve this goal is extremely advantageous, since it allows for the estimation of release kinetics before the creation of free release systems. Typically, the model is created by measuring several critical physical characteristics, such as the drug diffusion coefficient and experimental release data (Ilgin et al. 2019). Model-linked techniques, including zero-order, first-order, Hixson–Crowell, Higuchi, and Korsmeyer–Peppas models were utilized to examine the optimal drug release kinetic mechanism explaining the solution profile, and are summarized in Table 3.

**Table 3:** Kinetic models of CS/PVA irradiated hydrogel (50/50): drug-release rate constants, diffusion exponents, diffusion types, and regression values in diverse media

Drug loaded in CS/PVA gels (µg/mg polymer)	Releasing media	Kinetic models						
		Zero-order	First-order	Hixson-Crowell	Higuchi	Korsmeyer–Peppas		
		*r*^*2*^	*r*^*2*^	*r*^*2*^	*r*^*2*^	*k*	*n*	*r*^*2*^
11.4	pH 2.1; PBS	0.9015	0.7850	0.8355	0.9878	0.0175	0.61 ± 0.04	0.9782
11.0	pH 7.4; PBS	0.8656	0.7630	0.7968	0.9618	0.0084	0.63 ± 0.05	0.9630
11.0	pH 5.5; DI	0.9743	0.8675	0.9192	0.9818	0.0023	0.72 ± 0.01	0.9981

In a zero-order model, drug elimination is constant regardless of concentration, whereas, in a first-order model, drug elimination rises proportionately as concentration increases (Rungrod et al. 2021). The plot according to the zero-order and first-order equations showed not best fitted with r^2^ values obtained around 0.76 and 0.90 for amoxicillin release in PBS at both 2.1 and 7.4. Meanwhile, zero-order kinetics describes the process of constant drug release from a drug delivery system that corresponds to drug release in DI medium with an r^2^ value of up to 0.97. It is noted that the drug release mechanism in DI water is governed by the relaxing of polymeric chains and has a constant release rate regardless of the concentration of the drug.

Higuchi drug release is a diffusion method based on Fick's law, which proposes that matrix swelling and evaporation are minor or insignificant and have a square-root time dependency (Kumari & Meena 2021). The correlation coefficients obtained for the Hixon–Crowell model (0.79–0.91) were lower than those found for the Higuchi model, indicating that this model could not suit the release mechanism. The diffusion-controlled release was eventually discovered to be the primary mechanism of drug kinetics compared to a change in the surface area and diameter of particles.

To explore the drug release mechanism from hydrogel or exist in more than one sort of release phenomenon, the Korsmeyer–Peppas model is helpful. In this model, a range of parameters composing polymer swelling, erosion, matrix porosity, and drug diffusion rates in swelling systems was investigated (Ilgin et al. 2019). The reported literature reveals that if *n* < 0.45, solvent penetration into the hydrogels follows the Fickian process. Furthermore, if *n* is between 0.45 and 0.89, the drug release is controlled by diffusion and polymer network relaxation. This is referred to as a non-Fickian process. However, several *n* larger than 0.89 represents drug release as a function of polymer gel system expansion or relaxation. In this work, amoxicillin releases followed non-Fickian with n values from 0.61 to 0.72 for different pH environments and the *r*^2^ value applied for all conditions was greater than 0.95, as shown in Table 3. As a result, drug release occurs in response to both diffusion and swellable porous matrix.

## Conclusions

We synthesized and characterized hybrid hydrogels to precisely regulate drug release. Gamma irradiation was used as a green approach to generate hydrogels of high-molecular-weight CS and PVA without toxic chemicals. With an increase in the PVA concentration and radiation dose up to 25 kGy, the gel contents, thermal stabilities, mechanical strengths, and swelling degrees of the blended hydrogels significantly increased as compared to those of the neat PVA and CS hydrogels due to the increased generation of free radicals and formation of chemical bonds and the presence of the amino groups of CS. Nevertheless, when the radiation dose was increased to 30 kGy, a slightly weaker hydrogel was acquired owing to the possibility of CS chain scission. Therefore, the composite hydrogel produced under these conditions exhibited important drug release abilities due to its porous structure. Furthermore, the in vitro drug release studies revealed that the antibiotic drug release abilities of hydrogels were strongly dependent on the pH of the solutions and the releasing media. The data describing drug release were acquired using five well-known kinetic models, and the release profile was matched to the Higuchi and Korsmeyer–Peppas models. Non-Fickian diffusion is indicated by n values for the diffusion exponent in the Korsmeyer–Peppas model for swelling and drug release. It is proposed that the created gamma-irradiated CS/PVA hydrogels be employed as medication carriers.

## Data Availability

The data sets used and/or analysed during the current study are available from the corresponding author on reasonable request.
